# Prophylactic antibiotics for postcataract surgery endophthalmitis: a systematic review and network meta-analysis of 6.8 million eyes

**DOI:** 10.1038/s41598-022-21423-w

**Published:** 2022-10-18

**Authors:** Ai Kato, Nobuyuki Horita, Ho Namkoong, Eiichi Nomura, Nami Masuhara, Takeshi Kaneko, Nobuhisa Mizuki, Masaki Takeuchi

**Affiliations:** 1 Department of Ophthalmology and Visual Science, Yokohama City University Graduate School of Medicine, 3-9 Fukuura, Kanazawa-ku, Yokohama, 236-0004 Japan; 2 Department of Ophthalmology, Chigasaki Municipal Hospital, Chigasaki, Kanagawa Japan; 3 Chemotherapy Center, Yokohama City University Hospital, Yokohama, Kanagawa Japan; 4 Department of Infectious Diseases, Keio University School of Medicine, Shinjuku, Tokyo, Japan; 5 Department of Pulmonology, Yokohama City University Graduate School of Medicine, Yokohama, Kanagawa Japan

**Keywords:** Lens diseases, Eye diseases, Bacterial infection

## Abstract

To reveal optimal antibiotic prophylactic regimen for postoperative endophthalmitis (POE), we conducted systematic review and network meta-analysis. A total of 51 eligible original articles, including two randomized controlled trials, were identified. In total, 4502 POE cases occurred in 6,809,732 eyes (0.066%). Intracameral injection of vancomycin had the best preventive effect (odds ratio [OR] 0.03, 99.6% confidence interval [CI] 0.00–0.53, corrected P-value = 0.006, P-score = 0.945) followed by intracameral injection of cefazoline (OR 0.09, 99.6% CI 0.02–0.42, corrected P-value < 0.001, P-score = 0.821), cefuroxime (OR 0.18, 99.6% CI 0.09–0.35, corrected P-value < 0.001, P-score = 0.660), and moxifloxacin (OR 0.36, 99.6% CI 0.16–0.79, corrected P-value = 0.003, P-score = 0.455). While one randomized controlled trial supported each of intracameral cefuroxime and moxifloxacin, no randomized controlled trial evaluated vancomycin and cefazoline. Sensitivity analysis focusing on the administration route revealed that only intracameral injection (OR 0.19, 99.4% CI 0.12–0.30, corrected P-value < 0.001, P-score = 0.726) significantly decreased the risk of postoperative endophthalmitis. In conclusion, intracameral injection of either vancomycin, cefazoline, cefuroxime, or moxifloxacin prevented POE.

## Introduction

Cataract surgery is the most commonly performed ophthalmologic procedure in many industrialized countries, and its frequency continues to increase. An aging society and improved technologies are key factors augmenting the number of cataract surgeries^1^. Endophthalmitis is a sight-threatening disorder caused by intraocular infection through endogenous or exogenous routes. Postoperative endophthalmitis (POE) is a possible complication of intraocular surgeries, particularly cataract surgery, and it can lead to loss of vision. The most common cause of POE is bacteria from the eyelid flora^2,3^. Therefore, the use of perioperative antibiotics is a reasonable strategy for reducing the occurrence of POE. In daily practice, various antibiotics have been used to prevent endophthalmitis, and various routes of antibiotic administration have been proposed accordingly. However, the benefit of antibiotic use has not been sufficiently clear until recently. Because the incidence of postcataract surgery endophthalmitis is less than 0.1%, and it is difficult to design a randomized controlled trial (RCT) for such a rare complication. Therefore, our practice has relied largely on evidence from observational studies. A meta-analysis is also helpful in overcoming disease rarity^4^. Published pairwise meta-analyses revealed that perioperative intracameral vancomycin and moxifloxacin^5^, anterior chamber injection of moxifloxacin after cataract surgery^6^, and intracameral cefuroxime and moxifloxacin after cataract surgery^7^ reduced the risk of endophthalmitis compared with no antibiotic prophylaxis. However, each of these head-to-head meta-analyses evaluated limited treatment options only. Therefore, the optimal antibiotic type and administration route for endophthalmitis prevention are still not evident.

The current systematic review and network meta-analysis revealed the optimal antibiotic for preventing POE using data from both randomized controlled trials and observational studies.

## Results

### Study selection and characteristics

**Figure 1:**
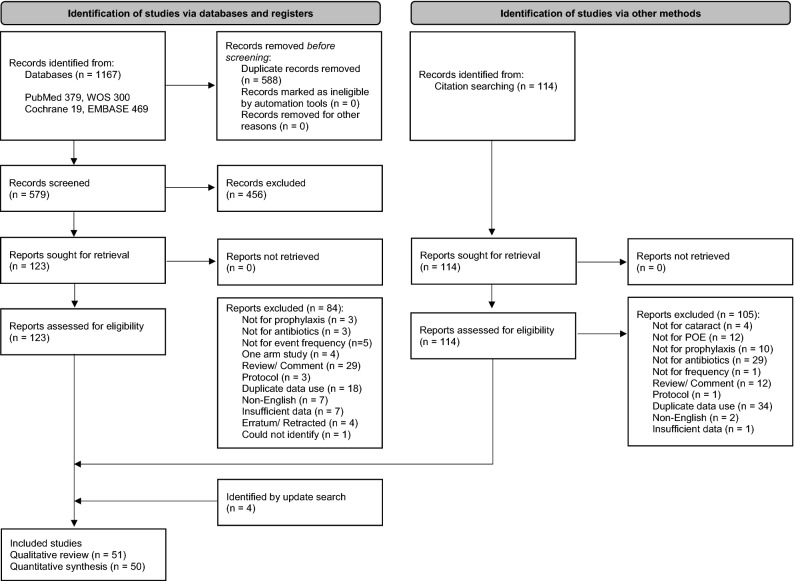
Preferred reporting items for systematic reviews and meta-analyses 2020 flow diagram. *POE* postoperative endophthalmitis.

We found a total of 1167 and 114 articles by database search and manual search, respectively (Fig. 1). After screening and full-text reading, 47 articles were found to be eligible for our analysis. Approximately 1 year later, the updated database search detected four additional studies. (Fig. 1, Supplementary Reference 1 and Supplementary Table 1).

**Figure 2:**
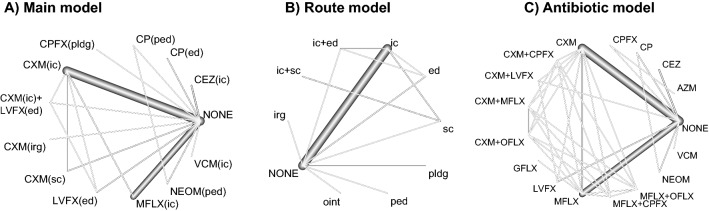
Network graphs. *NONE* no prophylactic antibiotics, *CEZ* cefazoline, *CXM* cefuroxime, *CAZ* ceftazidime, *MFLX* moxifloxacin, *CPFX* ciprofloxacin, *OFLX* ofloxacin, *GFLX* gatifloxacin, *LVFX* levofloxacin, *VCM* vancomycin, *NEOM* neomycin, *GM* gentamycin, *TB* tobramycin, *AZM* azithromycin, *CP* chloramphenicol, *VCM* vancomycin. *ic* intracameral, *ed* eye drop, *ped* pre-operative eye drop, *irg* irrigation, *sc* subconjunctival injection, *pldg* pledget.

Among the 51 studies, 43 studies were retrospective observational studies, 6 were prospective observational studies, and only 2 were RCTs (Table 1). The United States had the largest number of reports (N = 10), followed by Spain (N = 7), India (N = 6), Brazil (N = 3), China (N = 3), and Sweden (N = 3). While 45 studies provided data for head-to-head comparison in our study, 6 studies provided data for three or more treatment arms. Thirty-eight reports (75%) designed an arm without prophylactic antibiotics. Single-drug intracameral cefuroxime (N = 15) and single-drug intracameral moxifloxacin (N = 12) were the most widely used antibiotic regimens. The network plot also revealed that the comparison between no prophylactic antibiotic and these two regimens was evaluated most frequently (Fig. 2A).

**Table 1:** Characteristics of included studies.

Study	Country	Design	Eyes	NOS	Arm 1	Arm 2	Arm 3–8
Akkach (2019) ^8^	Australia	Retro	5900	7	NONE	CP(ed)	
Allen (1974) ^9^	USA	Retro	36,000	7	NONE	NEOM 0.5% (ped)	CP 0.4%(ped)
Anijeet (2010) ^10^	UK	Retro	16,606	7	NONE	VCM 1 mg/0.1 mL(ic)	
Asencio (2014) ^11^	Spain	Retro	14,285	7	GM 40 mg/0.5 mL(sc) + AG(ed)	GM 0.08% (irg) + VCM 0.1%(irg) + AG(ed)	
Barreau (2012) ^12^	France	Pro	5115	7	NONE	CXM(ic)	
Barry (2007) ^13^	Ireland	RCT	16,211	9	NONE	CXM(ic)	LVFX(ed) CXM(ic) + LVFX(ed)
Bhatta (2021)^14^	Nepal	Retro	111,983	7	MFLX 0.5 mg/0.1 mL(ic) + GM 20 mg/0.5 mL(sc)	GM 20 mg/0.5 mL(sc)	
Bohigian (2007) ^15^	USA	Retro	5268	7	NONE	CPFX(pldg)	
Cheng (2014) ^16^	Australia	Retro	99,448	7	NONE	CEZ(ic)	
Colleaux (2000) ^17^	Canada	Retro	13,886	7	NONE	ANY(sc)	
Daien (2016) ^18^	France	Retro	2,434,008	7	None	MFLX 0.5 mg/0.1 mL(ic)	
Dave (2021) ^19^	India	Retro	66,967	7	NONE	CXM(ic)	
Ferlini (2013) ^20^	Argentina	Retro	6001	7	NONE	MFLX 0.5 mg/0.1 mL(ic)	
Friling (2019) ^21^	Sweden	Retro	109,534	7	NONE	CXM 1 mg/mL(ic)	
Galvis (2014) ^22^	Colombia	Retro	2674	7	NONE	MFLX 0.25 mg/0.05 mL(ic)	
Garat (2009) ^23^	Spain	Retro	18,579	7	NONE	CEZ 2.5 mg/0.1 mL(ic)	
Garcia-S (2010) ^24^	Spain	Retro	13,652	7	NONE	CXM 1 mg/0.1 mL(ic)	
Guo (2021) ^25^	Australia	Retro	42,877	7	CP(ed)	NONE	
Haripriya (2019) ^26^	India	Retro	2,062,643	7	NONE	MFLX 0.5 mg/0.1 mL(ic)	
Hollander (2004) ^27^	USA	Retro	2718	7	NONE	ANY(oint)	
Jensen (2008) ^28^	USA	Retro	29,276	7	ANY(ed)	GFLX 0.3%(ed)	MFLX 0.5%(ed)
Katz (2015) ^29^	Israel	Retro	56,094	7	NONE	CXM(ic)	
Kingrey (2019) ^30^	USA	Retro	30,649	7	ANY(top)	ANY(top) + VCM(ic)	
Li (2018) ^31^	China	Retro	4210	7	NONE	CXM(ic)	
Li (2019) ^32^	USA	Retro	32,526	7	ANY(top)	ANY(ic)	
Lundström (2007) ^33^	Sweden	Pro	225,471	7	NONE	CXM(ic)	
Ma (2020) ^34^	China	Retro	61,299	7	NONE	CXM 0.03% (irg)	
Matsuura (2013) ^35^	Japan	Retro	34,752	7	NONE	MFLX 0.05–0.5 mg/mL(ic)	
Melega (2019) ^36^	Brazil	RCT	3640	9	NONE	MFLX 0.15 mg/0.03 mL (ic)	
Moser (2019) ^37^	Spain	Retro	55,984	7	OFLX 0.3%(top)	CEZ 2.4 mg/0.3 mL(ic) + OFLX 0.3%(top)	CEZ 2.4 mg/0.3 mL(ic) + MFLX(top)
Moshirfar (2007) ^38^	USA	Retro	20,013	7	GFLX(top)	MFLX(top)	
Paiva (2016) ^39^	Brazil	Retro	200	7	NONE	MFLX 150-μg/0.03 mL (ic)	
Porwal (2021) ^40^	India	Retro	19,853	7	NONE	CP 5 mg/mL(ed)	
Råen (2013) ^41^	Norway	Retro	15,254	7	NONE	MFLX(ic)	
Rahman (2015) ^42^	Ireland	Retro	16,975	7	NONE	CXM(ic)	
Rathi (2020) ^43^	India	Pro	42,466	7	CXM 1 mg/0.1 mL(ic)	CXM1mg/0.1 mL(ic) + CPFX(top)	CXM1mg/0.1 mL(ic) + OFLX(top) CXM1mg/0.1 mL(ic) + MFLX(top) MFLX 0.5 mg/0.1 mL(ic) MFLX 0.5 mg/0.1L(ic) + CPFX(top) MFLX 0.5 mg/0.1 mL(ic) + OFLX(top) MFLX 0.5 mg/0.1 mL(ic) + MFLX(top)
Rodriguez-C (2013) ^44^	Spain	Retro	19,463	7	NONE	CXM 1 mg/0.1 mL(ic)	
Romero-A (2012) ^45^	Spain	Pro	25,001	7	NONE	CEZ 1 mg/0.1 mL(ic)	
Rudnisky (2014) ^46^	Canada	Retro	75,295	7	NONE	MFLX(ic)	
Rush (2015) ^47^	USA	Retro	20,719	7	NONE	VCM 1 mg/0.1 mL(ic)	
Sharma (2015) ^48^	India	Pro	15,122	7	NONE	CXM 1 mg/0.1 mL(ic)	
Shenoy (2021) ^49^	India	Retro	214,782	7	NONE	MFLX 0.5 mg/0.1 mL(ic)	
Shorstein (2013) ^50^	USA	Retro	4916	7	NONE	CXM or MFLX 0.5 mg/0.1 mL, 2.5 mg/0.5 mL, 5 mg/mL(ic)	
Shorstein (2021) ^51^	USA	Retro	204,655	7	CXM 1 mg/0.1 mL(ic)	MFLX 0.1%(ic)	
Sobaci G (2009) ^52^	Turkey	Retro	6099	7	NONE	CXM(ic)	
Tan (2012) ^53^	Singapore	Retro	50,177	7	CEZ 1 mg/0.1 mL(sc) + GM 8 mg/0.2 mL(sc)	CEZ 1 mg/0.1 mL(ic)	
Tuñí-P (2018) ^54^	Spain	Retro	15,146	7	AZM(ed) 15 mg/g	CPFX(ed) 3 mg/ml	
Vieira (2017) ^55^	Brazil	Retro	7195	7	ANY(ed)	ANY(ed) + MFLX(ic) 0.27 mg/0.05 mL	
Wejde (2005) ^56^	Sweden	Pro	158,679	7	NONE	ANY(ic)	
Yao (2013) ^57^	China	Retro	201,757	7	VCM 1 mg/0.1 mL(ic), 1%(irg) + TB(sc/top)	TB (irg/sc/top)	
Yu-W-M (2008) ^58^	UK	Retro	37,170	7	NONE	CXM 1 mg/0.1 mL(ic)	CXM 50 mg/0.5 mL(sc)

RCT, randomized controlled trial; Pro, prospective observational study; Retro, retrospective observational study.

NOS, The Newcastle–Ottawa Scale.

NONE, no prophylactic antibiotics; CEZ, cefazoline; CXM, cefuroxime; CAZ, ceftazidime; MFLX, moxifloxacin; CPFX, ciprofloxacin; OFLX, ofloxacin; GFLX, gatifloxacin; LVFX, levofloxacin; VCM, vancomycin; NEOM, neomycin; AG, aminoglycoside; GM, gentamycin; TB, tobramycin; AZM, azithromycin; CP, Chloramphenicol.

(ic), intracameral; (ed), eye drop; (ped), pre-operative eye drop; (irg), irrigation; (oint), ointment; (sc), subconjunctival injection; (pldg), pledget; (top), topical.

The median number of eyes in a report was 25,001; however, the sample sizes varied greatly, ranging from 200 to 2,434,008. The total number of analyzed cases was 6,809,732, with 4502 endophthalmitis cases therein. Therefore, the raw event frequency was 0.066%.

Study quality was evaluated using the Newcastle–Ottawa Scale ranging from 7 to 9 (Table 1). The Cochrane Risk of Bias Summary was also presented for two RCTs as Supplementary Fig. 1. Although the straightforward clinical question of the current analysis made it easy to attain a high score on the scale, all observational studies provided only unadjusted raw numbers of patients with and without endophthalmitis.

A study by Asencio^11^ was not used for quantitative synthesis because of the use of unspecified aminoglycoside class agent and because it constituted an independent loop in the route model.

### Main analysis

Four eligible studies were excluded from the main analysis because they composited independent loops: Tuñí-P^54^, azithromycin eye drop versus moxifloxacin eye drop; Jensen^28^, gatifloxacin eye drop versus moxifloxacin eye drop; Asencio^11^, “aminoglycoside eyedrop plus subconjunctival gentamycin” versus “aminoglycoside eyedrop plus irrigation with vancomycin plus gentamycin”; Bhatta^14^, “moxifloxacin intracameral injection plus subconjunctival gentamycin” versus “subconjunctival gentamycin.”

The network plot is shown in Fig. 2A. Overall, a moderate inconsistency was observed (I^2^ = 82%). The total number of evaluated eyes was 6,141,523.

**Figure 3:**
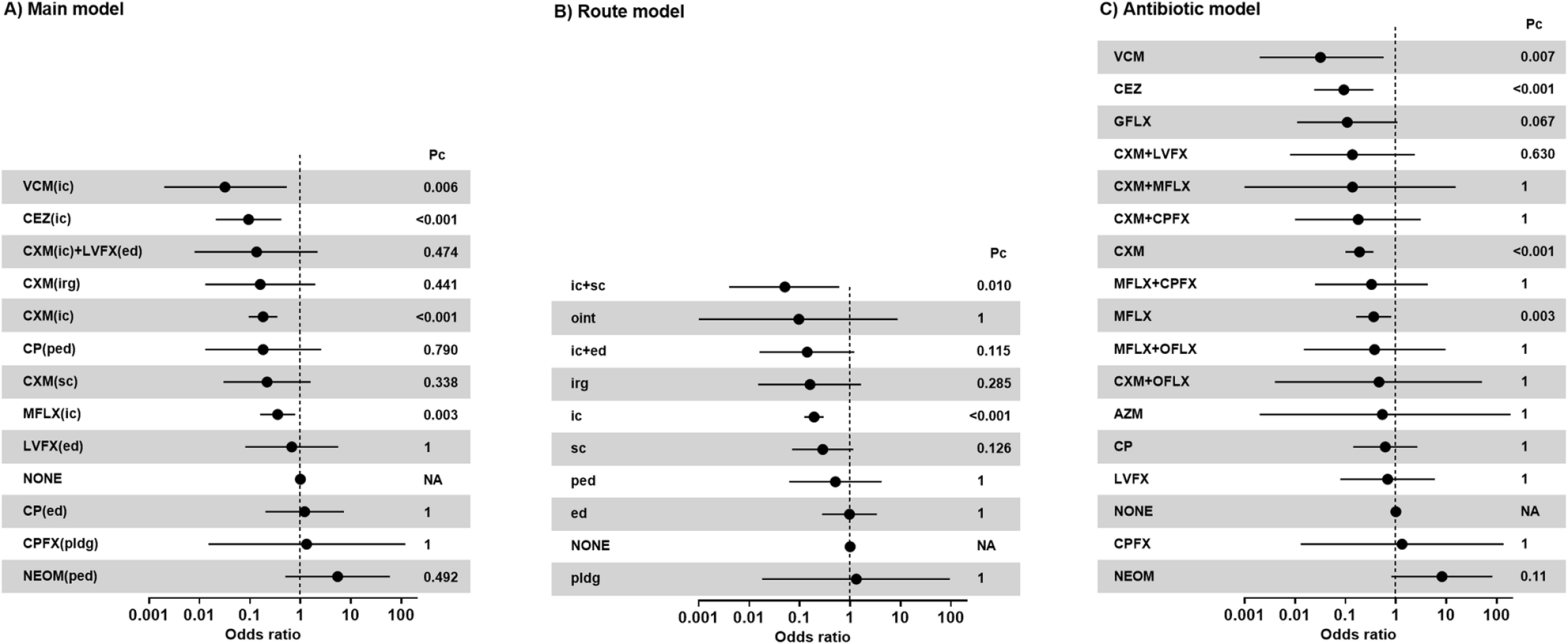
Forest plots. Common comparator was no antibiotic prophylaxis (NONE). Please note that 99.6% confidence interval (CI) for the main model, 99.4% CI for the route model, and 99.7% CI for the antibiotic model were used. Please see Supplementary Table 7 for more detail. Pc, Bonferroni-corrected P value. Please see Supplementary Table 7 for more detail. *NONE* no prophylactic antibiotics, *CEZ* cefazoline, *CXM* cefuroxime, *CAZ* ceftazidime, *MFLX* moxifloxacin, *CPFX* ciprofloxacin, *OFLX* ofloxacin, *GFLX* gatifloxacin, *LVFX* levofloxacin, *VCM* vancomycin, *NEOM* neomycin, *GM* gentamycin, *TB* tobramycin, *AZM* azithromycin, *CP* chloramphenicol, *VCM* vancomycin. *ic* intracameral, *ed* eye drop, *ped* pre-operative eye drop, *irg* irrigation, *sc* subconjunctival injection, *pldg* pledget.

A random-model network meta-analysis comprising of 36 reports revealed that intracameral vancomycin had the best preventive effect for endophthalmitis with an OR of 0.03 (99.6% CI 0.00–0.53, Pc = 0.006, Fig. 3A). This treatment also had the highest P-score (0.945) among the 12 treatment options (Supplementary Table 2). Other antibiotics prevented POE were cefazoline intracameral injection (OR 0.09, 99.6% CI 0.02–0.42, Pc < 0.001, P-score = 0.821), cefuroxime intracameral injection (OR 0.18, 99.6% CI 0.09–0.35, Pc < 0.001, P-score = 0.660), and intracameral moxifloxacin (OR 0.36, 99.6% CI 0.16–0.79, Pc = 0.003, P-score = 0.455) (Fig. 3A, Supplementary Table 2). Although some other regimens had lower OR values, these regimens did not have a significant prophylactic effect.

### Sensitivity analysis focusing on administration route

Data from 42 articles with 6,239,835 postsurgical eyes were analyzed for this route-comparison analysis. Intracameral injection was compared with no antibiotics in 31 studies, and this comparison involved the largest number of cases (Fig. 2B). According to this network meta-analysis, two intracameral injection-related regimens (intracameral + subconjunctival injection, OR 0.05, 99.4% CI 0.00–0.61, Pc = 0.010, P-score = 0.901; intracameral injection, OR 0.19, 99.4% CI 0.12–0.30, Pc < 0.001, P-score = 0.637) significantly decreased POE risk (Fig. 3B, Supplementary Table 3). Of note, the route with the lowest POE incidence and the highest P-score was intracameral + subconjunctival injection; however, Bhatta^14^ was the only study who evaluated this route. The intra-cameral antibiotic doses used in the various published literature were summarized in Table 1. The scatter plot for 31 studies that compared intracameral injection and no antibiotic arms suggests marginal possibility of weak publication bias (Kendall test tau = 0.22, P = 0.092 < 0.1, Supplementary Fig. 2). The use of ointment (OR 0.10, 99.4% CI 0.00–8.77, P-score = 0.720) and irrigation (OR 0.160, 99.4% CI 0.02–1.65, P-score = 0.668) resulted in lower OR values than intracameral injection; however, data for these administration routes are scarce (Fig. 3B).

### Sensitivity analysis focusing antibiotic type

An additional network meta-analysis was conducted to compare the antibiotic types as a sensitivity analysis. Two studies that made separate small loops were excluded from the analysis: Yao^57^ compared vancomycin plus tobramycin and tobramycin; Moser^37^ comparing three arms; ofloxacin, cefazoline plus ofloxacin, and cefazoline plus moxifloxacin. A random-model network meta-analysis incorporating 6,396,287 cases from 40 studies revealed that vancomycin was associated with the lowest ophthalmitis risk (OR 0.03, 99.7% CI 0.00–0.57, Pc = 0.007) and the highest P-score of 0.930 (Figs. 2C, 3C, Supplementary Table 4). Cefazoline (OR 0.09, 99.7% CI 0.02–0.36, Pc < 0.001), cefuroxime (OR 0.19, 99.7% CI 0.10–0.36, Pc < 0.001), and moxifloxacin (OR 0.36, 99.7% CI 0.16–0.81, Pc < 0.001) also prevented postsurgical endophthalmitis (Fig. 3C). No combination treatment decreased the risk of endophthalmitis in this model (Fig. 3C).

### Sensitivity analyses at single-arm-level incidence

The pooled arm-level endophthalmitis incidence among eyes without antibiotics was 0.082% (95% CI 0.079–0.085, Table 2). Intracameral injection of vancomycin (0.004%, 95%CI 0–0.024), cefazoline (0.011%, 95%CI 0.001–0.022), moxifloxacin (OR 0.019, 95% CI 0.017–0.021), and cefuroxime (OR 0.045, 95% CI 0.041–0.048) lowered the risk of endophthalmitis in patients compared with those who did not use prophylactic antibiotics.

**Table 2:** Pooled arm-level postoperative endophthalmitis frequency.

Antibiotic	Studies (RCTs)	Eyes	Pooled frequency (%)	95% CI (%)
VCM(ic)	2 (0)	22,088	0.004	0.000–0.024
CEZ(ic)	4 (0)	70,249	0.011	0.000–0.022
MFLX(ic)	12 (1)	1,573,724	0.019	0.017–0.021
CP(ed)	4 (0)	55,498	0.027	0.011–0.043
CXM(ic)	15 (1)	1,450,272	0.045	0.041–0.048
NONE	39 (2)	2,987,583	0.082	0.079–0.085

Frequency was pooled using generic inverse variance method.

95% CI: 95% confidence interval.

About two or more articles were presented for preventive option evaluation.

NONE, no prophylactic antibiotics; CEZ, cefazoline; CXM, cefuroxime; MFLX, moxifloxacin; VCM, vancomycin; CP, Chloramphenicol.

(ic), intracameral; (ed), eye drop.

## Discussion

We conducted the first network meta-analysis to comprehensively evaluate the efficacy of antibiotics administered for the prevention of POE in a population of 6.8 million eyes. This study highlighted the efficacy of intracameral injection of cefuroxime and moxifloxacin in the prevention of POE in cataract surgery. Network meta-analysis is an analytical method developed as an extension of pairwise meta-analysis and is useful when multiple interventions are present in a single subject^59^. Network meta-analysis allows us to estimate the relative effects of all interventions by comparing direct and indirect evidence.

Among a wide variety of techniques, intracameral administration is currently the most reliable prophylaxis procedure with accumulated evidence (Fig. 3A,B). Intracameral vancomycin and cefazoline injections led to the best OR and P-scores in the main model (Fig. 3A, Supplementary Table 2). Cefuroxime and moxifloxacin via intracameral injection with the next best OR were supported by more robust evidence from numerous studies including one RCT for each drug (Table 1, Fig. 3A). These findings were validated by sensitivity analysis (Fig. 3C). To date, intracameral cefuroxime and moxifloxacin have been frequently evaluated on this topic; however, vancomycin and cefazoline may work as superior preventive medications. Efficacy of antibiotics through other routes such as ointment, irrigation, and subconjunctival injection were unclear. Furthermore, combination regimen advantage could not be confirmed.

We detected only two RCTs on this topic during our search. The European Society of Cataract and Refractive Surgeons multicenter study published in 2007 applied a 2 by 2 factorial design for 16,603 patients^13^. The use of intracameral cefuroxime injection soon after surgery lowered the risk of POE, whereas topical perioperative levofloxacin did not^13^. In 2019, Melega et al. reported another RCT that recruited 3640 patients. Intracameral moxifloxacin injection reduced the occurrence of postcataract endophthalmitis. Two ultra-large-scale retrospective cohort studies also confirmed the effectiveness of prophylactic intracameral antibiotic administration. Daien et al. investigated data from more than 2 million subjects and concluded that intracameral cefuroxime injection reduced the frequency of POE^18^. According to a recent Indian observational study with 2 million postsurgical eyes, intracameral moxifloxacin lowered the incidence^26^. Based on these RCTs and large cohort studies, intracameral cefuroxime and moxifloxacin were used as first-choice prophylaxis for POE after cataract surgery.

On the other hand, according to our network meta-analysis, vancomycin and cefazoline inoculated into the anterior chamber might be a better choice. We would like to discuss the merit of vancomycin and cefazoline in terms of antibacterial spectrum. Lalwani et al. reported that coagulase-negative staphylococcus accounted for 50 of 73 (68.4%) eyes as causative microbe of endophthalmitis after cataract surgery^60^. Fisch et al. described that POE was most frequently caused by *Staphylococcus epidermidis* and that gram-negative organisms were rarely isolated from this population^61^. Cataracts are an age-related condition, and elderly patients with a history of hospitalization or previous antimicrobial therapy are likely to carry methicillin‐resistant *S. aureus* or methicillin‐resistant *S. epidermidis*. From the perspective of antimicrobial spectrum, it is easy to explain why vancomycin, which is effective for methicillin-resistant microbes, was highly effective in preventing endophthalmitis. Cefazoline is a narrow-spectrum first-generation cephalosporin. That is especially effective against gram-positive bacteria, and it may be an optimal agent to cover POE-causing microbes. Cefazoline is widely used to prevent wound infection in various surgeries involving skin incisions^62^. Moxifloxacin is a broad-spectrum antibiotic that covers gram-positive, gram-negative, and anaerobic bacteria^63^. However, moxifloxacin is usually not prescribed for the treatment of gram-positive coccus infections because more potent antibiotics with narrower coverage are preferred in terms of bacterial resistance^64^. In fact, recent studies reported that the frequency of coagulase-negative staphylococcus-resistant against moxifloxacin has increased to more than 50%^65,66^. Antibiotic agent for prevention of postcataract surgery should be carefully considered. Regarding the results of the network meta-analysis and the antimicrobial spectrum, vancomycin and cefazolin provided into the anterior chamber are attractive options. Nevertheless, vancomycin and cefazoline have been evaluated in a small number of observational studies and in far fewer patients than cefuroxime and moxifloxacin (Tables 1, 2). We hope that more research will evaluate intracameral vancomycin and cefazoline in the future.

Limitations of this study should be mentioned. First, a majority of the articles used for quantitative synthesis were observational studies without confounding factor adjustment (Table 1). The frequency of POE of 0.066% makes it very difficult to conduct high-quality RCTs and prospective studies. We also mentioned in the “limitation” that the study year might influence the antibiotic efficacy results. In addition to the prophylactic administration of antimicrobial agents, the development of postoperative endophthalmitis is also associated with underlying diseases, therapeutic agents, incision type, disinfection, and intraoperative complications. However, these factors were not considered in this study. Moreover, the registered protocol’s insufficient description, lack of inquiry for the missing data, inclusion of conference abstract, possibility of clustered endophthalmitis, and inconsistent results among pooled studies may be limitations of our study. Despite this limitation, our research integrated the currently available data using a solid methodology. Our data would help clinicians and researchers select prophylactic antimicrobial administration after cataract surgery.

In conclusion, we performed the first network meta-analyses using data of more than 6.8 million eyes to identify efficacious antibiotic regimens to prevent POE. Multiple analyses confirmed the advantages of single-agent intracameral antibiotic administration. Cumulative evidence suggests that intracameral injection of cefuroxime and moxifloxacin decreased endophthalmitis. Vancomycin and cefazoline injected into the anterior chamber may be a better option. Single-agent intracameral injection of either vancomycin, cefazoline, cefuroxime, or moxifloxacin prevented POE, and we hope that in future, more research will evaluate intracameral vancomycin and cefazoline in detail.

## Methods

### Overview

The protocol for this systematic review, which complied with the Preferred Reporting Items for Systematic Reviews and Meta-Analyses (PRISMA) statement (Supplementary Table 5) has been registered on the website of the University Hospitals Information Network (UMIN) Center (ID: UMIN000044376)^67^ [UMIN Center. UMIN Clinical Trials Registry. Available at https://www.umin.ac.jp/ctr/index.htm. Accessed on November 25, 2021].

No industry sponsor had any role in our study.

### Study search

The electronic database search formulas for PubMed, Web of Science Core Collection, Cochrane Advanced Search, and Embase were composited by well-experienced investigators and are listed in Supplementary Table 6. These databases were searched on May 28, 2021 without time period limitation. An explosion was used for the Embase. Additional manual searches were performed independently by two review authors (AK and MT). References in the review articles (Supplementary Reference 2) and eligible original articles (Table 1) were checked for this step. An updated database search on PubMed was conducted on July 22, 2022.

A review author (NH) provided the EndNote file of candidate articles from database for two review authors after duplication removal using EndNote function. At this step, an additional Excel file of the same list was made. The two review authors independently screened all studies in the file and marked potentially included articles on the Excel sheet. The two review authors read the full text of articles that were marked by at least one review author to eventually select eligible articles. When two authors could not resolve a disagreement, a third author participated in the discussion accordingly (NH).

### Publication type and trial design

Both published randomized controlled trials and observational studies were included as long as they were written in English language and if they provided sufficient data. A conference abstract was also permitted as part of the search outcome in this study.

### Patients

Patients who underwent cataract surgery were included in this study. Cataract surgery included phacoemulsification and aspiration, as well as intracapsular and extracapsular cataract extraction. The degree of cataract hardness, length and location of the incision wound, concurrent intraocular lens insertion, and intraoperative complications were not considered.

No exclusion criteria were set for age and comorbidities, such as diabetes and immune-compromised status.

### Treatments

Perioperative antibiotics that were administered to patients via any route were accepted. Prophylactic antibiotics were categorized with a combination of antibiotic type and administration route for the main analysis. Antibiotics type was grouped by generic names regardless of brand names. Administration routes were categorized into followings: intracameral injection, eye drop, pre-operative eye drop, irrigation, subconjunctival injection, ointment, and pledget. A combined regimen of two or more antibiotics was also allowed.

### Quality assessment

The Newcastle–Ottawa Quality Assessment Scale for cohort studies was used for quality assessment. This scale was originally designed for non-RCT studies. However, we used this scale for both RCT and observational studies because each item of the scale is applicable even for an RCT. The Cochrane Risk of Bias Tool was used to additionally evaluate the two RCTs.

### Data extraction

The two review authors (AK and MT) extracted the study characteristics, such as author name, year of publication, country of origin, study title, type of antibiotic, route of administration, and number of endophthalmitis events input into Excel sheet. The two review authors checked the original research papers together to solve this once there was a disagreement. When this process did not work well, a third review author (NH) made the decision.

### Primary outcome from the main analysis

In the main analysis, the frequencies of postcataract surgery endophthalmitis were compared in terms of odds ratio (OR) as the primary endpoint using a random-model network meta-analysis. Treatment arms were determined by the combination of antibiotic type and administration route, e.g., "intracameral cefuroxime injection". Non-specific categories were not included in the analysis. For example, "intracameral injection of any antibiotic" and "topical administration of vancomycin or moxifloxacin" were excluded. "Topical" administration was not allowed as a category since it was too vague.

### Secondary outcomes from the sensitivity analysis

The secondary outcomes were also the frequencies of postcataract surgery endophthalmitis compared and measured using OR. In the first sensitivity network meta-analysis, treatments were categorized by administration route regardless of antibiotic type (route model). The second sensitivity network meta-analysis compared antibiotics ignoring administration maneuvers (antibiotic model).

Treatment-level endophthalmitis frequencies were calculated using a generic variance meta-analysis. The Agresti and Coullb method was applied to estimate standard error^68^.

### Statistics

The proportion of eyes with endophthalmitis were compared between the two treatment groups using ORs. When one or more cells were null in a two-by-two contingency table, 0.5 was added to all cells as continuity correction. The logarithm of the ORs and their standard errors were pooled using frequentist weighted least squares approach random-model network meta-analysis by the "netmeta" package in R (Gerta Rucker, Freiburg, Germany)^69,70^. A no prophylactic antibiotic (NONE) arm was used as the common comparator, and each prophylactic arm was tested for comparison with the NONE arm. P-values in the network meta-analysis were Bonferroni-corrected to prevent an increase in alpha error due to multiple comparisons (corrected P value, Pc). Similarly, the alpha values for each analysis were reduced, and the confidence intervals were adjusted accordingly (Supplementary Table 7). Overall heterogeneity was assessed by the package. The P-score, which makes ranking order on a 0–1 scale, a frequentist analog to the surface under the cumulative ranking curve, was provided for each network meta-analysis model^71^.

I^2^ statistics used for heterogeneity evaluation was interpreted as: I^2^ = 0%, no heterogeneity; I^2^ > 0% and < 30%, minimal heterogeneity; I^2^ ≥ 30% and < 60%, mild heterogeneity; I^2^ ≥ 60% and < 85%, moderate heterogeneity; and I^2^ ≥ 85%, considerable heterogeneity.

## Supplementary Information

Supplementary Information.

## Data Availability

The data supporting the findings of this study are available from the corresponding author upon reasonable request.
