# Supplementary material for: Prophylactic antibiotics for postcataract surgery endophthalmitis: a systematic review and network meta-analysis of 6.8 million eyes
Source: Sci Rep. 2022 Oct 18;12:17416. doi: 10.1038/s41598-022-21423-w (PMC9579149; doi:10.1038/s41598-022-21423-w)

**Prophylactic antibiotics for postcataract surgery** **endophthalmitis: a systematic review and network meta-analysis of 6.8 million eyes.**

**Ai Kato et al.**

**Yokohama City University**

**Supplement File**

**Supplementary Table 1. References excluded by the full-text reading.**

**Supplementary Table 2. P-score for the main model.**

**Supplementary Table 3. P-score for the route model sensitivity analysis.**

**Supplementary Table 4. P-score for antibiotic model sensitivity analysis.**

**Supplementary Table 5. PRISMA 2020 Checklist.**

**Supplementary Table 6. Search formulas.**

**Supplementary Table 7. Corrected alpha and confidence interval.**

**Supplementary References 1. List of finally eligible articles.**

**Supplementary References 2. List of hand searched review articles.**

**Supplementary Figure 1. Cochrane Risk of Bias for randomized controlled studies.**

**Supplementary Figure 2. Funnel plot for the comparison between intracameral injection and no prophylactic antibiotics.**

**Supplementary Table 1. References excluded by the full-text reading.**

| References from database | Reason |
| --- | --- |
| Akkach. Clin Exp Ophthalmol. 2018;46:53-54. | Duplicate date use |
| Allardice. J Hosp Infect. 2001;49(1):23-29. | Not for frequency |
| Allen. Trans Am Acad Ophthalmol Otolaryngol. 1973;77(5):op581-588. | Duplicate date use |
| Anijeet. Ophthalmology. 2010;117(4):853-853.e851. | Review/Comment |
| Aonuma. Jpn J Ophthalmol. 1999;53(5):815-818. | Non-English |
| Apt. Br J Ophthalmol. 1994;78(12):948-949. | Review/Comment |
| Arshinoff. J Cataract Refract Surg. 2008;34(12):2006-2008. | Review/Comment |
| Arshinoff. J Cataract Refract Surg. 2011;37(12):2105-2114. | Review/Comment |
| Barry. J Cataract Refract Surg. 2006;32(3):407-410. | Duplicate date use |
| Barry. J Cataract Refract Surg. 2014;40(1):138-142. | Insufficient data |
| Bellini. Br J Ophthalmol. 2011;95(6):892-893. | One study arm |
| Camesasca. Eur J Ophthalmol. 2008;18(3). | Erratum/Retracted |
| Cao. PLoS ONE. 2013;8(8). | Review/Comment |
| Cetinkaya. Int Eye Science. 2015;15(10):1680-1683. | Not for frequency |
| Chen. Eye. 2011;25(4):530-531. | Review/Comment |
| Clinicalrials.gov.Identifiler: NCT00136344. 2005 | Protocol |
| Creuzot-Garcher. Ophthalmology. 2016;123(7):1414-1420. | Insufficient data |
| Daien. Invest Ophthalmol Vis Sci. 2016;57(12):1990. | Duplicate date use |
| de Geus. Ophthalmol Retina. 2020. | Not for prophylaxis |
| Ding. Infection. 2011;39(5):451-460. | One arm study |
| Diwan. Invest Ophthalmol Vis Sci. 2010;51(13). | Could not identify |
| Egger. Spektrum Der Augenheilkunde. 2007;21(2):104-106. | Non-English |
| Egger. Spektrum Der Augenheilkunde. 2016;30(4-5):166-168. | Review/Comment |
| Euctr. http://wwwwhoint/trialsearch/Trial2aspx?TrialID=EUCTR2009-014054-14-GB. 2010. | Protocol |
| Fernandez Lopez. Eur J Hosp Pharm. 2014;21:A126. | Review/Comment |
| Friling. J Cataract Refract Surg. 2013;39(1):15-21. | Duplicate date use |
| Garat. J Cataract Refract Surg. 2005;31(11):2230-2234. | Duplicate date use |
| García-Sáenz. Arch Soc Esp Oftalmol. 2010;85(8):263-267. | Duplicate date use |
| Ghosh. Clin Exp Ophthalmol. 2014;42:32. | Not for frequency |
| Gimbel. Eur J Implant Refract Surg. 1994;6(5):280-285. | One arm study |
| Grasso. Evidence-Based Ophthalmology. 2008;9(2):118-119. | Duplicate date use |
| Grzybowski. Curr Pharm Des. 2017;23(4):565-573. | Review/Comment |
| Gualino. J Francais d'Ophtalmologie. 2010;33(8):551-555. | Non-English |
| Han. Clin Exp Ophthalmol. 2020;48(4):539-540. | Review/Comment |
| Haripriya. Asia Pac J Ophthalmol (Phila). 2017;6(4):324-329. | Review/Comment |
| Jabbarvand. Ophthalmology. 2016;123(2):295-301. | Erratum/Retracted |
| Kohnen. J Cataract Refract Surg. 2009;35(4):609. | Review/Comment |
| Krummenauer. Pharmacoepidemiol Drug Saf. 2006;15(9):662-666. | Erratum/Retracted |
| Leung. Int Ophthalmol Clinics. 2020;60(4):113-126. | Review/Comment |
| Leyngold. Cochrane Database of Systematic Reviews. 2007(1). | Review/Comment |
| Li. Eye contact lens. 2020;46(2):126-126. | Erratum/Retracted |
| Lloyd. Can J Ophthalmol. 2009;44(3):288-292. | Insufficient data |
| Lundström. J Cataract Refract Surg. 2015;41(11):2410-2416. | Not for prophylaxis |
| Ma. Infect Drug Resist. 2020;13:1455-1463. | Duplicate date use |
| Melega. J Cataract Refract Surg. 2019;45(3):343-350. | Duplicate date use |
| Meyer. Clin Exp Ophthalmol. 2016;44(7):643-645. | Not for antibiotics |
| Montan. J Cataract Refract Surg. 2002;28(6):977-981. | One study arm |
| Montan. Ophthalmology. 1998;105(12):2171-2177. | Not for antibiotics |
| Morgan. Can J Ophthalmol. 2000;35(7):378-378. | Review/Comment |
| Moser. Graefes Arch Clin Exp Ophthalmol. 2019;257(10):2185-2191. | Non-English |
| Nagar. Cochrane Database Syst Rev. 2007(3). | Review/Comment |
| Nagar. Cochrane Database Syst Rev. 2013;2013(5). | Protocol |
| Ng. J Cataract Refract Surg. 2007;33(2):269-280. | Not for porophylaxis |
| Nguyen. Clin Exp Ophthalmol. 2016;44:82. | Review/Comment |
| Nowak. Int J Environ Res Public Health. 2019;16(12). | Not for antibiotics |
| Pijl. Eye. 2011;25(4):530. | Review/Comment |
| Raen. Acta Ophthalmologica. 2013;91(2):118-122. | Duplicate date use |
| Raizman. Archives of Ophthalmology. 2011;129(4):501-502. | Review/Comment |
| Rana. Clin Exp Ophthalmol. 2021;49(1):25-37. | Review/Comment |
| Rathi. Invest Ophthalmol Vis Sci. 2019;60(9). | Duplicate date use |
| Rekas. Value in Health Regional Issues. 2020;22:115-121. | Insufficient data |
| Ricardo Suárez. Revista Mexicana de Oftalmologia. 2017;91(2):67-72. | Non-English |
| Romero. J Cataract Refract Surg. 2006;32(3):438-441. | Duplicate date use |
| Romero. J Cataract Refract Surg. 2006;32(3):438-441. | Duplicate date use |
| Romero-Aroca. Bmc Ophthalmology. 2012;12. | Duplicate date use |
| Rosha. Clin Exp Ophthalmol. 2006;34(6):535-544. | Not for frequency |
| Schmitz. Ophthalmology. 1999;106(10):1869-1877. | Insufficient data |
| Seal. J Cataract Refract Surg. 2006;32(3):396-406. | Duplicate date use |
| Seal. J Cataract Refract Surg. 2006;32(3):396-406. | Duplicate date use |
| Shimizu. Jpn J Ophthalmol. 1997;51(2):211-214. | Non-English |
| Simina. Rom J Ophthalmol. 2021;65(1):2-9. | Review/Comment |
| Sun. Infect Drug Resist. 2021;14:1231-1238. | Insufficient data |
| Surgeons. J Cataract Refract Surg. 2007;33(6):978-988. | Duplicate date use |
| Tan. Graefes Arch Clin Exp Ophthalmol. 2016;254(7):1437-1438. | Review/Comment |
| Tan. J Cataract Refract Surg. 2015;41(5):1125-1126. | Review/Comment |
| Titiyal. Indian J Ophthalmol. 2020;68(5):688-691. | Review/Comment |
| Verstappen. J Francais D Ophtalmologie. 2017;40(3):E103-E104. | Non-English |
| Wang. J Ophthalmol. 2020;2020. | Review/Comment |
| Wani. JK Practitioner. 2004;11(2):130-132. | Review/Comment |
| Wejde. J Hosp Infect. 2005;61(3):251-256. | Not for frequency |
| Williamson. Adv Ophthalmol Optom. 2018;3(1):407-421. | Review/Comment |
| Wu. International Eye Science. 2015;15(10):1753-1756. | Review/Comment |
| Zagaria. Us Pharmacist. 2016;41(4):8-+. | Review/Comment |
| Zhu. Scientific Reports. 2017;7. | Insufficient date |
| References from manual searching | Reason |
| Al-Mezaine. J Cataract Refract Surg. 2009;35(4):643-649. | Not for prophylaxis |
| Anijeet. Clin Ophthalmol. 2010;4:321-326. | Duplicate date use |
| Bainbridge. Br J Ophthalmol. 1998;82(11):1312-1315. | Not for antibiotics |
| Balent. Ophthalmic Surg Lasers. 2001;32:446-455. | Not for antibiotics |
| Barreau. J Cataract Refract Surg. 2012;38(8):1370-1375. | Duplicate date use |
| Barry. J Cataract Refract Surg. 2006;32(3):407-410. | Duplicate date use |
| Barry. J Cataract Refract Surg. 2006;32(3):407-410. | Duplicate date use |
| Benoist d'Azy. PLoS One. 2016;11(6):e0156431. | Not for cataract |
| Cao. PLoS One. 2013;8(8):e71731. | Review/Comment |
| Cataract Management Guideline Panel.US Department of Health and Human Services Agency for Health Care Policy and Research. AHCPR Publication. 1993;93-0541: | Protocol |
| Cetinkay. International Eye Science. 2015;15:1680-1683. | Duplicate date use |
| Chalmers. BMJ. 1992;305:786-8. | Not for prophylaxis |
| Colleaux. Can J Ophthalmol. 2000;35(7):373-378. | Duplicate date use |
| Colleaux. Can J Ophthalmol. 2000;35(7):373-378. | Duplicate date use |
| Cooper. Am J Ophthalmol. 2003;136(2):300-305. | Not for antibiotics |
| Coskun. J Ocul Pharmacol Ther. 2011;27:589-592. | Not for POE |
| Das. Ophthalmol. 2012;298459 | Not for prophylaxis |
| Deng. J Cataract Refract Surg. 2013;39:497-500. | Not for antibiotics |
| Doft.Arch Ophthalmo. 2001;119(5):650-656. | Not for prophylaxis |
| Ellis. Clin Experiment Ophthalmol. 2003;31(2):125-128. | Not for antibiotics |
| Endophthalmitis Study Group. J Cataract Refract Surg. 2007;33:978-988. | Duplicate date use |
| Endophthalmitis Study Group. J Cataract Refract Surg. 2007;33:978-988. | Duplicate date use |
| ESCRS. J Cataract Refract Surg. 2007;3:978-988. | Duplicate date use |
| Evidence-Based Medicine Working Group. JAMA. 1992;268: 2420-5. | Not for prophylaxis |
| Fileta. Ophthalmic Surg Lasers Imaging Retina. 2014;8(8):e71731. | Not for cataract |
| Freeman. Arch Ophthalmol. 2010;128(2):230-234. | Not for antibiotics |
| Friling. J Cataract Refract Surg. 2013;39:15-21. | Duplicate date use |
| Garat. J Cataract Refract Surg. 2009;35(4):637-642. | Duplicate date use |
| García Collado. Aten Farm. 2008;10(1):44-47. | Non-English |
| Garcia-Arumi. J Cataract Refract Surg. 2007;33(6):989-992. | Not for prophylaxis |
| García-Sáenz. Arch Soc Esp Oftalmol. 2010;85(8):263-267. | Non-English |
| García-Sáenz. J Cataract Refract Surg. 2010;36:203-207. | Duplicate date use |
| Halachmi-Eyal. J Cataract Refract Surg. 2009;35:2109-2114. | Not for POE |
| Haripriya. J Cataract Refract Surg. 2012;38:1360-1369. | Not for antibiotics |
| Haripriya. J Cataract Refract Surg. 2019;45:1226-1233. | Duplicate date use |
| Haripriya. Ophthalmology.2016;123:302-308. | Duplicate date use |
| Hatch. Ophthalmology. 2009;116(3):425-430. | Not for prophylaxis |
| He. J Ocul Pharmacol Ther. 2009;25:373-378. | Not for POE |
| Hennig. Br J Ophthalmol. 2003;87:266-270. | Not for antibiotics |
| Huang. PLoS One. 2016;11(11):e0166141. | Review/Comment |
| Hughes. Br J Ophthalmol. 1994;78:227-32. | Review/Comment |
| Inoue. Jpn J Ophthalmol. 2008;52:151-161. | Not for POE |
| Jabbarvand. Ophthalmology. 2016;123:295-301. | Duplicate date use |
| Javitt. Arch Ophthalmol. 1991;109(8):1085-1089. | Duplicate date use |
| Javitt. Arch Ophthalmol. 1991;109(8):1085-1089. | Duplicate date use |
| Javitt. Ophthalmology. 1994;100:100-6. | Not for prophylaxis |
| Kalpadakis. Eur J Ophthalmol. 2002;12(5):395-400. | Not for antibiotics |
| Kamalarajah. Eye (Lond). 2007;21(5):580-586. | Not for antibiotics |
| Keay. Ophthalmology. 2012;119(5):914-922. | Not for antbiotics |
| Khanna. BMJ Open. 2012;2:e001035. | Not for antibiotics |
| Lalitha. Ophthalmology. 2005;112(11):1884-1889. | Not for antibiotics |
| Lane. J Cataract Refract Surg . 2008;34(9):1451-1459. | Not for POE |
| Lertsumitkul. Ophthalmol. 2001;29(6):400-405. | Not for antibiotics |
| Li. Invest Ophthalmol Vis Sci. 2004;45(5):1321-1328. | Not for antibiotics |
| Li. J Cataract Refract Surg. 2015;41:724-731. | Not for POE |
| Linertová. Clin Ophthalmol. 2014;14(8):1515-22. | Review/Comment |
| Lloyd. Can J Ophthalmol. 2009;44(3):288-292. | Duplicate date use |
| Lundström. Ophthalmology. 2007;114(5):866-870. | Duplicate date use |
| Matsuura. J Cataract Refract Surg . 2013;39(11):1702-1706. | Duplicate date use |
| Matsuura. J Ocul Pharmacol Ther. 2014;30(9):771-776. | Not for POE |
| Mayer. Br J Ophthalmol. 2003;87(7):867-869. | Not for antibiotics |
| McCannel. Retina. 2011;31(4):654-61. | Not for cataract |
| McCarty. Br J Ophthalmol. 1997;81(2): 97-8. | Review/Comment |
| Melega. J Cataract Refract Surg. 2019;45(3):343-350. | Duplicate date use |
| Menchini. Eye (Lond). 2018;32(9):1423-1431. | Review/Comment |
| Miño de Kaspar. Am J Ophthalmol. 2008;145:136-142. | Not for POE |
| Mohan. Indian J Ophthalmol. 2017;65:1477-1482. | Not for antibiotics |
| Montan. Acta Ophthalmol Scand. 2002;80(3):258-261. | Duplicate date use |
| Montan. J Cataract Refract Surg. 2002;28:977-981. | Not for frequency |
| Moss. Ophthalmol. 2008;115:2013-2016. | Not for POE |
| Nagaki. J Cataract Refract Surg. 2003;29(1):20-26. | Not for antibiotics |
| Ng. J Cataract Refract Surg. 2007;33(2):269-280. | Duplicate date use |
| Norregaard. Br J Ophthalmol. 1997;81(2):102-106. | Not for antibiotics |
| Park. J Cataract Refract Surg. 2021;48(1):100-112. | Review/Comment |
| Pleyer. Klin Monbl Augenheilkd. 2008;225(11):934-940. | Review/Comment |
| Rana. Clin Exp Ophthalmol. 2021;49:25-37. | Duplicate date use |
| Ravindran. J Cataract Refract Surg. 2009;35:629-636. | Not for antibiotics |
| Romero-Aroca. BMC Ophthalmol. 2012;12:2. | Duplicate date use |
| Rudnisky. Ophthalmology. 2014;121(4):835-841. | Duplicate date use |
| Rudnisky. Ophthalmology. 2014;121(4):835-841. | Duplicate date use |
| Schmitz. Ophthalmology. 1999;106(10):1869-1877. | Duplicate date use |
| Sharifi. Ophthalmology. 2009;116:1887-1896. | Review/Comment |
| Shorstein. J Cataract Refract Surg. 2013;39(2):313. | Review/Comment |
| Shorstein. J Cataract Refract Surg. 2013;39: 8-14. | Duplicate date use |
| Steinberg. Health Serv Res. 1990;25:727-31. | Not for cataract |
| Swaddiwudhipong. J Med Assoc Thai. 2000;83(8):902-907. | Not for antibiotics |
| Ta. Eur J Ophthalmol. 2007;17:689-695. | Not for POE |
| Ta. J Ocul Pharmacol Ther. 2008;24:427-431. | Not for POE |
| Ta. Ophthalmol. 2002;109:2036-2040. | Not for POE |
| Taban. Arch Ophthalmol. 2005;123(5):613-20. | Review/Comment |
| Tan. J Cataract Refract Surg. 2012;38:425-430. | Duplicate date use |
| Venkatesh. Br J Ophthalmol. 2005;89:1079-1083. | Not for antibiotics |
| Vieira. Arquivos brasileiros de oftalmologia. 2017;80:165-167. | Duplicate date use |
| Virgilio. Ophthalmology and Eye Diseases. 2014;6: 1-4. | Duplicate date use |
| Wallin. J Cataract Refract Surg. 2005;31(4):735-741. | Not for prophylaxis |
| Wang. Ophthalmol. 2020;Article ID:7242969. | Review/Comment |
| Wejde. Acta Ophthalmol Scand. 2005;83(1): 7-10. | Insufficient data |
| Wejde. J Hosp Infect. 2005;61(3):251-256. | Duplicate date use |
| West. Ophthalmology. 2005;112(8):1388-1394. | Not for antibiotics |
| Wong. J Ophthalmol. 2004;88(1):29-31. | Not for antibiotics |
| Wong. Ophthalmology. 2004;111(4):699-705. | Not for antibiotics |
| Wu. J Ocul Pharmacol Ther. 2006;22(1):54-61. | Not for antibiotics |
| Yu-Wai-Man. J Cataract Refract Surg. 2008;34:447-451. | Duplicate date use |
| Zaman. J Ayub Med Coll Abbottabad. 2009;21:39-42. | Not for antibiotics |
| Zawar. Eur J Ophthalmol. 2011;21:748-753. | Not for antibiotics |
| Zeng. Nepal J Ophthalmol. 20A2:A10714;6:140-144. | Not for antibiotics |

**Supplementary Table 2. P-score for the main model.**

|  | P-score |
| --- | --- |
| VCM(ic) | 0.9450 |
| CEZ(ic) | 0.8206 |
| CXM(ic)+LVFX(ed) | 0.7129 |
| CXM(irg) | 0.6752 |
| CXM(ic) | 0.6604 |
| CP(ped) | 0.6425 |
| CXM(sc) | 0.5992 |
| MFLX(ic) | 0.4548 |
| LVFX(ed) | 0.3125 |
| CPFX(pldg) | 0.2460 |
| NONE | 0.2130 |
| CP(ed) | 0.1894 |
| NEOM(ped) | 0.0286 |

NONE, no prophylactic antibiotics; CEZ, cefazoline; CXM, cefuroxime; MFLX, moxifloxacin; CPFX, ciprofloxacin; LVFX, levofloxacin; NEOM, neomycin; CP, Chloramphenicol; VCM, vancomycin.

ic, intracameral; ed, eye drop; ped, pre-operative eye drop; irg, irrigation; sc, subconjunctival injection; pldg, pledget.

**Supplementary Table 3. P-score for the route model sensitivity analysis.**

|  | P-score |
| --- | --- |
| ic+sc | 0.9011 |
| oint | 0.7202 |
| ic+ed | 0.7062 |
| irg | 0.6680 |
| ic | 0.6372 |
| sc | 0.5017 |
| ped | 0.3481 |
| pldg | 0.2009 |
| ed | 0.1672 |
| NONE | 0.1494 |

ic, intracameral; ed, eye drop; ped, pre-operative eye drop; irg, irrigation; sc, subconjunctival injection; pld, pledget.

**Supplementary Table 4. P-score for antibiotic model sensitivity analysis.**

|  | P-score |
| --- | --- |
| VCM | 0.9298 |
| CEZ | 0.8111 |
| GFLX | 0.7607 |
| CXM+LVFX | 0.6998 |
| CXM+MFLX | 0.6645 |
| CXM | 0.6450 |
| CXM+CPFX | 0.6428 |
| MFLX+CPFX | 0.4881 |
| MFLX+OFLX | 0.4571 |
| MFLX | 0.4542 |
| CXM+OFLX | 0.4293 |
| AZM | 0.4249 |
| CP | 0.3248 |
| LVFX | 0.3070 |
| CPFX | 0.2407 |
| NONE | 0.1988 |
| NEOM | 0.0215 |

NONE, no prophylactic antibiotics; CEZ, cefazoline; CXM, cefuroxime; MFLX, moxifloxacin; CPFX, ciprofloxacin; OFLX, ofloxacin; LVFX, levofloxacin; NEOM, neomycin; AZM, azithromycin; CP, chloramphenicol; VCM, vancomycin.

**Supplementary Table 5. PRISMA 2020 Checklist.**

| **Section and Topic** | **Item #** | **Checklist item** | **Location where item is reported** |
| --- | --- | --- | --- |
| **TITLE** | | |  |
| Title | 1 | Identify the report as a systematic review. | Title |
| **ABSTRACT** | | |  |
| Abstract | 2 | See the PRISMA 2020 for Abstracts checklist. | Abstract |
| **INTRODUCTION** | | |  |
| Rationale | 3 | Describe the rationale for the review in the context of existing knowledge. | Introduction, Paragraph 1 |
| Objectives | 4 | Provide an explicit statement of the objective(s) or question(s) the review addresses. | Introduction, Paragraph 1 |
| **METHODS** | | |  |
| Eligibility criteria | 5 | Specify the inclusion and exclusion criteria for the review and how studies were grouped for the syntheses. | Methods, Study search |
| Information sources | 6 | Specify all databases, registers, websites, organisations, reference lists and other sources searched or consulted to identify studies. Specify the date when each source was last searched or consulted. | Methods, Study search |
| Search strategy | 7 | Present the full search strategies for all databases, registers and websites, including any filters and limits used. | Methods, Study search |
| Selection process | 8 | Specify the methods used to decide whether a study met the inclusion criteria of the review, including how many reviewers screened each record and each report retrieved, whether they worked independently, and if applicable, details of automation tools used in the process. | Methods, Study search |
| Data collection process | 9 | Specify the methods used to collect data from reports, including how many reviewers collected data from each report, whether they worked independently, any processes for obtaining or confirming data from study investigators, and if applicable, details of automation tools used in the process. | Methods, Study search |
| Data items | 10a | List and define all outcomes for which data were sought. Specify whether all results that were compatible with each outcome domain in each study were sought (e.g. for all measures, time points, analyses), and if not, the methods used to decide which results to collect. | Methods, Study search |
|  | 10b | List and define all other variables for which data were sought (e.g. participant and intervention characteristics, funding sources). Describe any assumptions made about any missing or unclear information. | Methods, Study search |
| Study risk of bias assessment | 11 | Specify the methods used to assess risk of bias in the included studies, including details of the tool(s) used, how many reviewers assessed each study and whether they worked independently, and if applicable, details of automation tools used in the process. | Methods, Study search |
| Effect measures | 12 | Specify for each outcome the effect measure(s) (e.g. risk ratio, mean difference) used in the synthesis or presentation of results. | Methods, Main analysis |
| Synthesis methods | 13a | Describe the processes used to decide which studies were eligible for each synthesis (e.g. tabulating the study intervention characteristics and comparing against the planned groups for each synthesis (item #5)). | Methods, Study search |
|  | 13b | Describe any methods required to prepare the data for presentation or synthesis, such as handling of missing summary statistics, or data conversions. | Methods, Study search |
|  | 13c | Describe any methods used to tabulate or visually display results of individual studies and syntheses. | Methods, Statistics |
|  | 13d | Describe any methods used to synthesize results and provide a rationale for the choice(s). If meta-analysis was performed, describe the model(s), method(s) to identify the presence and extent of statistical heterogeneity, and software package(s) used. | Methods, Statistics |
|  | 13e | Describe any methods used to explore possible causes of heterogeneity among study results (e.g. subgroup analysis, meta-regression). | Methods, Statistics |
|  | 13f | Describe any sensitivity analyses conducted to assess robustness of the synthesized results. | Methods, Sensitivity analysis |
| Reporting bias assessment | 14 | Describe any methods used to assess risk of bias due to missing results in a synthesis (arising from reporting biases). | Methods, Statistics |
| Certainty assessment | 15 | Describe any methods used to assess certainty (or confidence) in the body of evidence for an outcome. | Methods, Statistics |
| **RESULTS** | | |  |
| Study selection | 16a | Describe the results of the search and selection process, from the number of records identified in the search to the number of studies included in the review, ideally using a flow diagram. | Results, Study selection and characteristics |
|  | 16b | Cite studies that might appear to meet the inclusion criteria, but which were excluded, and explain why they were excluded. | Results, Study selection and characteristics |
| Study characteristics | 17 | Cite each included study and present its characteristics. | Results, Study selection and characteristics |
| Risk of bias in studies | 18 | Present assessments of risk of bias for each included study. | Supplementary material |
| Results of individual studies | 19 | For all outcomes, present, for each study: (a) summary statistics for each group (where appropriate) and (b) an effect estimate and its precision (e.g. confidence/credible interval), ideally using structured tables or plots. | Table 1, Figure 2 |
| Results of syntheses | 20a | For each synthesis, briefly summarise the characteristics and risk of bias among contributing studies. | Results, Main analysis |
|  | 20b | Present results of all statistical syntheses conducted. If meta-analysis was done, present for each the summary estimate and its precision (e.g. confidence/credible interval) and measures of statistical heterogeneity. If comparing groups, describe the direction of the effect. | Results, Main analysis |
|  | 20c | Present results of all investigations of possible causes of heterogeneity among study results. | Results, Main analysis |
|  | 20d | Present results of all sensitivity analyses conducted to assess the robustness of the synthesized results. | Results, Sensitivity analysis |
| Reporting biases | 21 | Present assessments of risk of bias due to missing results (arising from reporting biases) for each synthesis assessed. | Supplementary material |
| Certainty of evidence | 22 | Present assessments of certainty (or confidence) in the body of evidence for each outcome assessed. | Supplementary material |
| **DISCUSSION** | | |  |
| Discussion | 23a | Provide a general interpretation of the results in the context of other evidence. | Discussion, Paragraph 1-3 |
|  | 23b | Discuss any limitations of the evidence included in the review. | Discussion, Paragraph 4 |
|  | 23c | Discuss any limitations of the review processes used. | Discussion, Paragraph 4 |
|  | 23d | Discuss implications of the results for practice, policy, and future research. | Discussion, Paragraph 6 |
| **OTHER INFORMATION** | | |  |
| Registration and protocol | 24a | Provide registration information for the review, including register name and registration number, or state that the review was not registered. | Methods, Protocol registration |
|  | 24b | Indicate where the review protocol can be accessed, or state that a protocol was not prepared. | Methods, Protocol registration |
|  | 24c | Describe and explain any amendments to information provided at registration or in the protocol. | N/A |
| Support | 25 | Describe sources of financial or non-financial support for the review, and the role of the funders or sponsors in the review. | Funding |
| Competing interests | 26 | Declare any competing interests of review authors. | Competing interests |
| Availability of data, code and other materials | 27 | Report which of the following are publicly available and where they can be found: template data collection forms; data extracted from included studies; data used for all analyses; analytic code; any other materials used in the review. | Supplementary material |

*From:*  Page MJ, McKenzie JE, Bossuyt PM, Boutron I, Hoffmann TC, Mulrow CD, et al. The PRISMA 2020 statement: an updated guideline for reporting systematic reviews. BMJ 2021;372:n71. doi: 10.1136/bmj.n71

For more information, visit: <http://www.prisma-statement.org/>

From: Stroup DF, Berlin JA, Morton SC, et al, for the Meta-analysis Of Observational Studies in Epidemiology (MOOSE) Group. Meta-analysis of Observational Studies in Epidemiology. A Proposal for Reporting. JAMA. 2000;283(15):2008-2012. doi: 10.1001/jama.283.15.2008.

**Supplementary Table 6. Search formulas.**

| PubMed |  |
| --- | --- |
| (endophthalmitis [title] OR endophthalmia [title]) (cataract OR phacoemulsification) (prophyla* OR prophylactic OR prophylaxis OR antibioti* OR antibiotic OR antibiotics OR cefazoline OR cefuroxime OR ceftazidime OR moxifloxacin OR ciprofloxacin OR ofloxacin OR gatifloxacin OR levofloxacin OR vancomycin OR gentamycin OR azithromycin) | 379 |
| Web of Science |  |
| #1 TI=((endophthalmitis OR endophthalmia) AND (cataract OR phacoemulsification))  #2 TS=(prophylactic OR prophylaxis OR antibiotic OR antibiotics OR cefazoline OR cefuroxime OR ceftazidime OR moxifloxacin OR ciprofloxacin OR ofloxacin OR gatifloxacin OR levofloxacin OR vancomycin OR gentamycin OR azithromycin)  #3 #1 AND #2 | 300 |
| Cochrane Central |  |
| (endophthalmitis:ti OR endophthalmia:ti) AND (cataract:ti OR phacoemulsification:ti) | 19 |
| EMBASE |  |
| (prophylactic OR 'prophylaxis'/exp OR prophylaxis OR 'antibiotic'/exp OR antibiotic OR 'antibiotics'/exp OR antibiotics OR 'cefazoline'/exp OR cefazoline OR 'cefuroxime'/exp OR cefuroxime OR 'ceftazidime'/exp OR ceftazidime OR 'moxifloxacin'/exp OR moxifloxacin OR 'ciprofloxacin'/exp OR ciprofloxacin OR 'ofloxacin'/exp OR ofloxacin OR 'gatifloxacin'/exp OR gatifloxacin OR 'levofloxacin'/exp OR levofloxacin OR 'vancomycin'/exp OR vancomycin OR 'gentamycin'/exp OR gentamycin OR 'azithromycin'/exp OR azithromycin) AND (endophthalmitis:ti OR endophthalmia:ti) AND (cataract:ti OR phacoemulsification:ti) | 469 |

Databases were searched on 28^th^, 2021.

**Supplementary Table 7. Corrected alpha and confidence interval.**

| Model | N of arm | Correction factor | Alpha | Confidence interval |
| --- | --- | --- | --- | --- |
| Main model | 13 | 12 | 0.0042 | 99.6 |
| Route model | 10 | 9 | 0.0055 | 99.4 |
| Antibiotics model | 17 | 16 | 0.0031 | 99.7 |

Correction factor was determined by "number (N) of arms - 1." This is because each of prophylaxes in each model were compared to no-treatment (NONE) arm and tested.

Alpha was determined by "0.05/correction factor."

Bonferroni-corrected P value (Pc) was determined by multiplying raw P value by the correction factor

Confidence interval was determined in accordance with corresponding alpha value.

**Supplementary References 1. List of finally eligible articles.**

1. Akkach S, Kam J, Meusemann R. Post-cataract surgery endophthalmitis: The role of prophylactic antibiotic eye drops. Clin Exp Ophthalmol. 2019;47(4):555-556.
2. Allen HF, Mangiaracine AB. Bacterial endophthalmitis after cataract extraction. II. Incidence in 36,000 consecutive operations with special reference to preoperative topical antibiotics. Archives of ophthalmology (Chicago, Ill : 1960). 1974;91(1):3-7.
3. Anijeet DR, Palimar P, Peckar CO. Intracameral vancomycin following cataract surgery: An eleven-year study. Clin Ophthalmol. 2010;4:321-326.
4. Asencio MA, Huertas M, Carranza R, Tenias JM, Celis J, Gonzalez-del Valle F. Impact of changes in antibiotic prophylaxis on postoperative endophthalmitis in a Spanish hospital. Ophthalmic Epidemiol. 2014;21(1):45-50.
5. Barreau G, Mounier M, Marin B, Adenis JP, Robert PY. Intracameral cefuroxime injection at the end of cataract surgery to reduce the incidence of endophthalmitis: French study. J Cataract Refract Surg. 2012;38(8):1370-1375.
6. Bhatta, S., Pant, N. & Poudel, M. Postoperative endophthalmitis with and without intracameral moxifloxacin prophylaxis in a high volume surgery setting. *BMJ open ophthalmology*. 2021;**6:** e000609.
7. Prophylaxis of postoperative endophthalmitis following cataract surgery: results of the ESCRS multicenter study and identification of risk factors. J Cataract Refract Surg. 2007;33(6):978-988.
8. Bohigian GM. A retrospective study of the incidence of culture-positive endophthalmitis after cataract surgery and the use of preoperative antibiotics. Ophthalmic Surg Lasers Imaging. 2007;38(2):103-106.
9. Cheng N, Kam J, Dawkins R, Sandhu S, Allen P. Post-cataract surgery endophthalmitis in the modern era: Can we do better? Clinical and Experimental Ophthalmology. 2014;42:33.
10. Colleaux KM, Hamilton WK. Effect of prophylactic antibiotics and incision type on the incidence of endophthalmitis after cataract surgery. Can J Ophthalmol. 2000;35(7):373-378.
11. Daien V, Papinaud L, Gillies MC, et al. Effectiveness and Safety of an Intracameral Injection of Cefuroxime for the Prevention of Endophthalmitis After Cataract Surgery With or Without Perioperative Capsular Rupture. JAMA Ophthalmol. 2016;134(7):810-816.
12. Dave VP, Singh VM, Reddy JC, et al. Clinical features and microbiology of post-cataract surgery endophthalmitis with and without intracameral moxifloxacin prophylaxis: Endophthalmitis prophylaxis study report 3. Indian J Ophthalmol. 2022;70:158-163.
13. Ferlini L, Perrone L, Caride GG, et al. Intracameral moxifloxacin for prophylaxis of endophthalmitis after cataract surgery: A case Series. Investigative Ophthalmology & Visual Science. 2013;54(15).
14. Friling E, Montan P. Bacteriology and cefuroxime resistance in endophthalmitis following cataract surgery before and after the introduction of prophylactic intracameral cefuroxime: a retrospective single-centre study. J Hosp Infect. 2019;101(1):88-92.
15. Galvis V, Tello A, Sánchez MA, Camacho PA. Cohort study of intracameral moxifloxacin in postoperative endophthalmitis prophylaxis. Ophthalmol Eye Dis. 2014;6:1-4.
16. Garat M, Moser CL, Martin-Baranera M, Alonso-Tarres C, Alvarez-Rubio L. Prophylactic intracameral cefazolin after cataract surgery: endophthalmitis risk reduction and safety results in a 6-year study. J Cataract Refract Surg. 2009;35(4):637-642.
17. García-Sáenz MC, Arias-Puente A, Rodríguez-Caravaca G, Bañuelos JB. Effectiveness of intracameral cefuroxime in preventing endophthalmitis after cataract surgery Ten-year comparative study. J Cataract Refract Surg. 2010;36(2):203-207.
18. Guo, B., Au, B., Allen, P. & Van Heerden, A. Role of chloramphenicol eye drops for endophthalmitis prophylaxis following cataract surgery: Outcomes of institutional cessation. Clin Exp Ophthalmol. 2021:49;1116-1118.
19. Haripriya A, Chang DF, Ravindran RD. Endophthalmitis reduction with intracameral moxifloxacin in eyes with and without surgical complications: Results from 2 million consecutive cataract surgeries. J Cataract Refract Surg. 2019;45(9):1226-1233.
20. Hollander DA, Stewart JM, Seiff SR. The role of pre-operative topical antibiotics in the prophylaxis of bacterial endophthalmitis post cataract surgery. Investigative Ophthalmology & Visual Science. 2004;45:U159-U159.
21. Jensen MK, Fiscella RG, Moshirfar M, Mooney B. Third- and fourth-generation fluoroquinolones: retrospective comparison of endophthalmitis after cataract surgery performed over 10 years. J Cataract Refract Surg. 2008;34(9):1460-1467.
22. Katz G, Blum S, Leeva O, et al. Intracameral cefuroxime and the incidence of post-cataract endophthalmitis: an Israeli experience. Graefes Arch Clin Exp Ophthalmol. 2015;253(10):1729-1733.
23. Kingrey B, Kingrey D. Incidence of endophthalmitis in cataract surgery with and without intracameral vancomycin, a clinical review of 30,649 cases of a single surgeon. Investigative Ophthalmology & Visual Science. 2019;60(9).
24. Li ZB, Zhou TX, Sun K, Su H, Bi WM, Zeng BQ. Evaluation of intracameral cefuroxime injection for endophthalmitis prophylaxis following phacoemulsification. International Eye Science. 2018;18(12):2282-2284.
25. Li A, Shao J, Gans R, Bena J, Goshe J. Postoperative Endophthalmitis Before and After Preferred Utilization of Prophylactic Intracameral Antibiotics for Phacoemulsification Cataract Surgeries at Cole Eye Institute. Eye & contact lens. 2019;45(5):306-309.
26. Lundstrom M, Wejde G, Stenevi U, Thorburn W, Montan P. Endophthalmitis after cataract surgery: a nationwide prospective study evaluating incidence in relation to incision type and location. Ophthalmology. 2007;114(5):866-870.
27. Ma X, Xie L, Huang Y. Intraoperative Cefuroxime Irrigation Prophylaxis for Acute-Onset Endophthalmitis After Phacoemulsification Surgery. Infect Drug Resist. 2020;13:1455-1463.
28. Matsuura K, Miyoshi T, Suto C, Akura J, Inoue Y. Efficacy and safety of prophylactic intracameral moxifloxacin injection in Japan. J Cataract Refract Surg. 2013;39(11):1702-1706.
29. Melega MV, Alves M, Cavalcanti Lira RP, et al. Safety and efficacy of intracameral moxifloxacin for prevention of post-cataract endophthalmitis: Randomized controlled clinical trial. J Cataract Refract Surg. 2019;45(3):343-350.
30. Moser CL, Lecumberri Lopez M, Garat M, Martin-Baranera M. Prophylactic intracameral cefazolin and postoperative topical moxifloxacin after cataract surgery: endophthalmitis risk reduction and safety results in a 16-year study. Graefes Arch Clin Exp Ophthalmol. 2019;257(10):2185-2191.
31. Moshirfar M, Feiz V, Vitale AT, Wegelin JA, Basavanthappa S, Wolsey DH. Endophthalmitis after uncomplicated cataract surgery with the use of fourth-generation fluoroquinolones: a retrospective observational case series. Ophthalmology. 2007;114(4):686-691.
32. De Paiva Lucena N, Ferreira KSA, Dos Santos BMA, Lynch MI, Lira RPC. Is Intracameral moxifloxacin a safe option for prevention of post cataract endophthalmitis? Investigative Ophthalmology and Visual Science. 2016;57(12):5403.
33. Porwal, A. C., Patel, A., Mathew, B. C. & Jethani, J. N. Incidence of postoperative endophthalmitis with and without use of intracameral moxifloxacin. Indian J Ophthalmol. 2021:69;1353-1354.
34. Råen M, Sandvik GF, Drolsum L. Endophthalmitis following cataract surgery: the role of prophylactic postoperative chloramphenicol eye drops. Acta Ophthalmol. 2013;91(2):118-122.
35. Rahman N, Murphy CC. Impact of intracameral cefuroxime on the incidence of postoperative endophthalmitis following cataract surgery in Ireland. Ir J Med Sci. 2015;184(2):395-398.
36. Rathi VM, Sharma S, Das T, Khanna RC. Endophthalmitis Prophylaxis Study, Report 2: Intracameral antibiotic prophylaxis with or without postoperative topical antibiotic in cataract surgery. Indian J Ophthalmol. 2020;68(11):2451-2455.
37. Rodríguez-Caravaca G, García-Sáenz MC, Villar-Del-Campo MC, Andrés-Alba Y, Arias-Puente A. Incidence of endophthalmitis and impact of prophylaxis with cefuroxime on cataract surgery. J Cataract Refract Surg. 2013;39(9):1399-1403.
38. Romero-Aroca P, Mendez-Marin I, Salvat-Serra M, Fernandez-Ballart J, Almena-Garcia M, Reyes-Torres J. Results at seven years after the use of intracamerular cefazolin as an endophthalmitis prophylaxis in cataract surgery. BMC Ophthalmol. 2012;12:2.
39. Rudnisky CJ, Wan D, Weis E. Antibiotic choice for the prophylaxis of post-cataract extraction endophthalmitis. Ophthalmology. 2014;121(4):835-841.
40. Rush SW, Vu D, Rush RB. The Safety and Efficacy of Routine Administration of Intracameral Vancomycin during Cataract Surgery. J Ophthalmol. 2015;2015:813697.
41. Sharma S, Sahu SK, Dhillon V, Das S, Rath S. Reevaluating intracameral cefuroxime as a prophylaxis against endophthalmitis after cataract surgery in India. J Cataract Refract Surg. 2015;41(2):393-399.
42. Shenoy P, Goh EJH, Kashikar R, et al. Impact of prophylactic intracameral moxifloxacin on post-cataract surgery endophthalmitis: data from a tertiary eye care facility in rural India. Int Ophthalmol. 2021;41(8):2729-2736.
43. Shorstein NH, Winthrop KL, Herrinton LJ. Decreased postoperative endophthalmitis rate after institution of intracameral antibiotics in a Northern California eye department. J Cataract Refract Surg. 2013;39(1):8-14.
44. Shorstein NH, Liu L, Carolan JA, Herrinton L. Endophthalmitis Prophylaxis Failures in Patients Injected With Intracameral Antibiotic During Cataract Surgery. Am J Ophthalmol. 2021;227:166-172.
45. Sobaci G, Uysal Y, Mutlu FM, Bayer A, Gungor R, Karagul S. Prophylactic usage of intracameral cefuroxime in the prevention of postoperative endophthalmitis. Int J Ophthalmol. 2009;9(8):1439-1443.
46. Tan CS, Wong HK, Yang FP. Epidemiology of postoperative endophthalmitis in an Asian population: 11-year incidence and effect of intracameral antibiotic agents. J Cataract Refract Surg. 2012;38(3):425-430.
47. Tuñí-Picado J, Martínez-Palmer A, Fernández-Sala X, et al. Infectious postoperative endophthalmitis after cataract surgery performed over 7 years. The role of azithromycin versus ciprofloxacin eye drops. Rev Esp Quimioter. 2018;31(6):15-21.
48. Vieira IV, Boianovsky C, Saraiva TJ, Godoy RB, Lake J. Safety and efficacy of intracameral moxifloxacin injection for prophylaxis of endophthalmitis after phacoemulsification. Arq Bras Oftalmol. 2017;80(3):165-167.
49. Wejde G, Montan P, Lundström M, Stenevi U, Thorburn W. Endophthalmitis following cataract surgery in Sweden: national prospective survey 1999-2001. Acta Ophthalmol Scand. 2005;83(1):7-10.
50. Yao K, Zhu Y, Zhu Z, et al. The incidence of postoperative endophthalmitis after cataract surgery in China: a multicenter investigation of 2006-2011. Br J Ophthalmol. 2013;97(10):1312-1317.
51. Yu-Wai-Man P, Morgan SJ, Hildreth AJ, Steel DH, Allen D. Efficacy of intracameral and subconjunctival cefuroxime in preventing endophthalmitis after cataract surgery. J Cataract Refract Surg. 2008;34(3):447-451.

**Supplementary References 2. List of hand searched review articles.**

1. McCarty C. Endophthalmitis following cataract extraction: the need for a systematic review of the literature. Br J Ophthalmol. 1997;81(2):97-98.

2. Taban M, Behrens A, Newcomb RL, et al. Acute endophthalmitis following cataract surgery: a systematic review of the literature. Arch Ophthalmol. 2005;123(5):613-620.

3. McCannel CA. Meta-analysis of endophthalmitis after intravitreal injection of anti-vascular endothelial growth factor agents: causative organisms and possible prevention strategies. Retina. 2011;31(4):654-661.

4. Cao H, Zhang L, Li L, Lo S. Risk factors for acute endophthalmitis following cataract surgery: a systematic review and meta-analysis. PLoS One. 2013;8(8):e71731.

5. Fileta JB, Scott IU, Flynn HW, Jr. Meta-analysis of infectious endophthalmitis after intravitreal injection of anti-vascular endothelial growth factor agents. Ophthalmic Surg Lasers Imaging Retina. 2014;45(2):143-149.

6. Linertova R, Abreu-Gonzalez R, Garcia-Perez L, et al. Intracameral cefuroxime and moxifloxacin used as endophthalmitis prophylaxis after cataract surgery: systematic review of effectiveness and cost-effectiveness. Clin Ophthalmol. 2014;8:1515-1522.

7. Benoist d'Azy C, Pereira B, Naughton G, Chiambaretta F, Dutheil F. Antibioprophylaxis in Prevention of Endophthalmitis in Intravitreal Injection: A Systematic Review and Meta-Analysis. PLoS One. 2016;11(6):e0156431.

8. Huang J, Wang X, Chen X, Song Q, Liu W, Lu L. Perioperative Antibiotics to Prevent Acute Endophthalmitis after Ophthalmic Surgery: A Systematic Review and Meta-Analysis. PLoS One. 2016;11(11):e0166141.

9. Menchini F, Toneatto G, Miele A, Donati S, Lanzetta P, Virgili G. Antibiotic prophylaxis for preventing endophthalmitis after intravitreal injection: a systematic review. Eye (Lond). 2018;32(9):1423-1431.

10. Wang XL, Huang XY, Wang Z, Sun W. The Anterior Chamber Injection of Moxifloxacin Injection to Prevent Endophthalmitis after Cataract Surgery: A Meta-analysis. J Ophthalmol. 2020;2020:7242969.

11. Park J, Popovic MM, Balas M, El-Defrawy SR, Alaei R, Kertes PJ. Clinical features of endophthalmitis clusters after cataract surgery and practical recommendations to mitigate risk: systematic review. Journal of cataract and refractive surgery. 2022;48(1):100-112.

12. Rana K, Bahrami B, van Zyl L, Esterman A, Goggin M. Efficacy of intracameral antibiotics following manual small incision cataract surgery in reducing the rates of endophthalmitis: A meta-analysis. Clin Exp Ophthalmol. 2021;49(1):25-37.

**Supplementary Figure 1. Cochrane Risk of Bias for randomized controlled studies.**

**
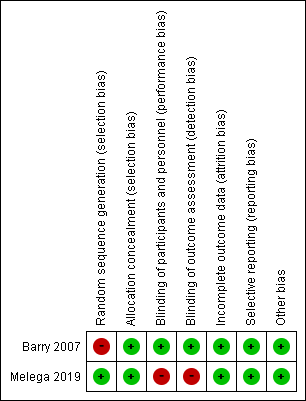
**

**Supplementary Figure 2. Funnel plot for the comparison between intracameral injection and no prophylactic antibiotics.**


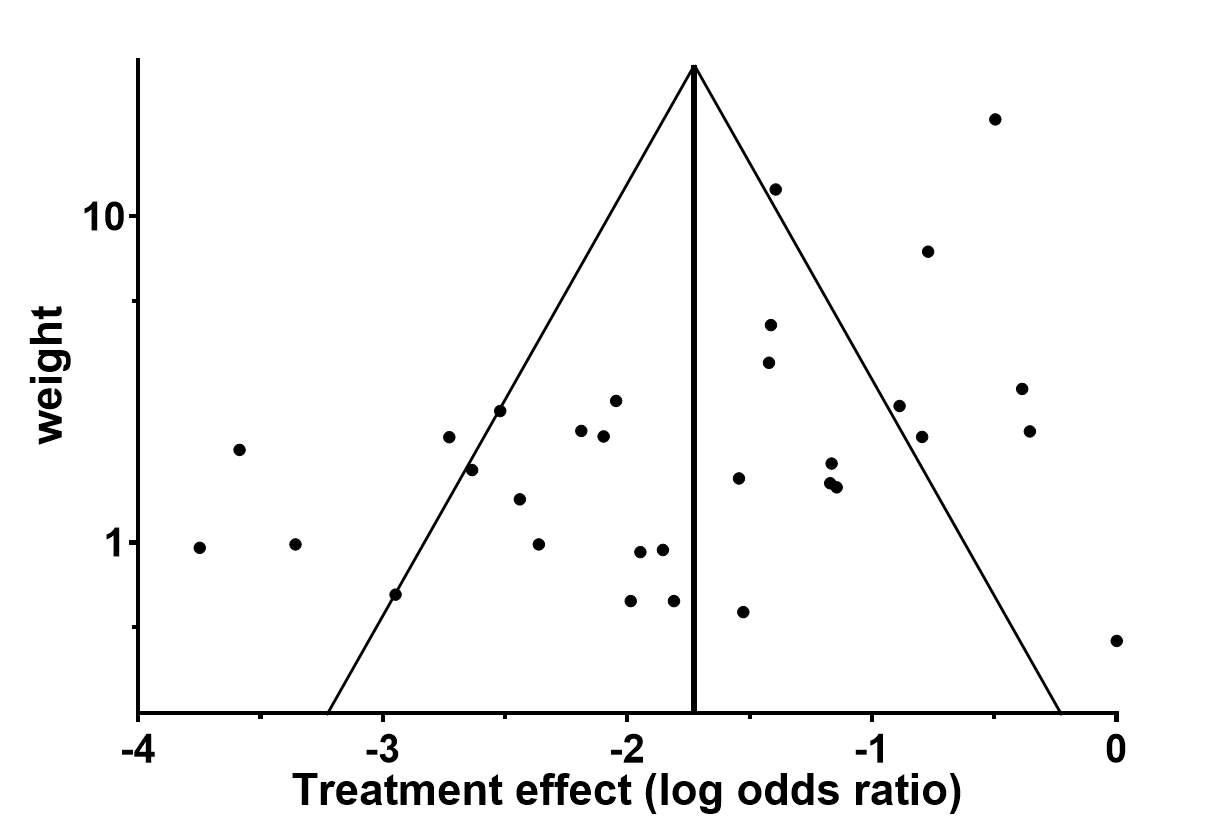

Supplement: Supplementary file 1 — Supplementary Information. [file 41598_2022_21423_MOESM1_ESM.docx]
